# A Cheap Filter

**Published:** 1890-06

**Authors:** 


### A CHEAP FILTER.

Among the most active causes of disease is impure water. Hence, unless good spring water can be obtained, that which is used for drinking should be filtered. No complicated and expensive filter is needed ; a very good one can be made out of a common earthenware flower-pot. This is an old fashioned device. Get a new flower pot with a hole in the bottom ; line it with a piece of new cotton flannel ; put into the bottom of this, to the depth of two inches, some clean sand ; over this put a layer of pounded charcoal, and fill up with fine gravel. This domestic filter will answer the purpose of the most elaborate, and it is very easily made. After several weeks' use it should be renewed.

